# Sleep Duration, Exercise, Shift Work and Polycystic Ovarian Syndrome-Related Outcomes in a Healthy Population: A Cross-Sectional Study

**DOI:** 10.1371/journal.pone.0167048

**Published:** 2016-11-21

**Authors:** Audrey J. R. Lim, Zhongwei Huang, Seok Eng Chua, Michael S. Kramer, Eu-Leong Yong

**Affiliations:** 1 Department of Obstetrics and Gynecology, National University Hospital, National University of Singapore, Singapore, 119228, Singapore; 2 Departments of Epidemiology, Biostatistics & Occupational Health and of Pediatrics, McGill University Faculty of Medicine, Montreal, Quebec, H3G1Y6, Canada; University of Rome Tor Vergata, ITALY

## Abstract

**Context:**

Few studies have examined the associations between sleep duration, shiftwork, and exercise to the infrequent menstruation, hyperandrogenism, and ovarian morphological changes observed in women with polycystic ovarian syndrome (PCOS).

**Objective:**

To examine whether lifestyle factors, including short sleep duration, insufficient exercise, and shiftwork, alone or in combination, are associated with the reproductive and metabolic abnormalities typical of PCOS in a healthy population.

**Study Design, Size, Duration:**

Prospective cross-sectional study of 231 women, including healthcare workers recruited for an annual health screen, healthy referral patients from the Women’s Clinic and volunteers from the university community at the National University Hospital, Singapore, from 2011 to 2015.

**Main Outcome Measures:**

The women completed a questionnaire, including their menstrual cycle length, sleep length, frequency of exercise and shift work. Hyperandrogenism (hirsutism score, testosterone, sex hormone binding globulin (SHBG)), ovarian morphology and function (anthral follicle count, ovarian volume, anti-mullerian hormone (AMH)), and metabolic measures (body mass index (BMI), waist hip ratio (WHR), blood pressure, fasting glucose, fasting insulin and fasting lipids) were examined through anthropometric measurements, transvaginal ultrasound scans, and blood tests.

**Results:**

No significant associations were observed between shift work, exercise or sleep duration and the androgenic and ovarian measures that define PCOS. However, women reporting fewer than 6 hours of sleep were more likely to report abnormal (short or long) menstrual cycle lengths (OR = 2.1; 95% CI, 1.1 to 4.2). Women who reported fewer than 6 hours of sleep had increased fasting insulin levels (difference in means = 2.13; 95% CI, 0.27 to 3.99 mU/L) and higher odds of insulin resistance (OR = 2.58; CI, 1.16 to 5.76). Lack of regular exercise was associated with higher mean fasting insulin (difference in means = 2.3 mU/L; 95% CI, 0.5 to 4.1) and HOMA-IR (difference in means = 0.49; 95% CI, 0.09 to 0.90) levels.

**Conclusions:**

Women with insufficient sleep are at increased risk of menstrual disturbances and insulin resistance, but do not have the hyperandrogenism and polycystic ovarian morphology typical of PCOS.

**Wider Implications of the Findings:**

Improved sleep duration may help reduce the risks of diabetes or infertility. Shift work, exercise or sleep duration appear not to impact the androgenic and ovarian measures that define PCOS.

## Introduction

The typical phenotype of polycystic ovarian syndrome (PCOS) is characterized by the presence of infrequent menstruation (>35 days for cycle length, or less than 8 cycles a year), hyperandrogenism (manifesting as hirsutism and/or elevated serum androgens), and polycystic ovarian morphology (PCOM) on ultrasound assessment. However as defined by the current diagnostic criteria for PCOS [1], numerous sub-phenotypes of the full syndrome are possible. At least three sub-phenotypes are generally recognized—oligomenorrhea and hyperandrogenism; PCOM and oligomenorrhea; PCOM and hyperandrogenism [2]. It is unclear whether these sub-phenotypes have distinct etiologies, or are merely precursors to the complete phenotype comprising of all three abnormalities.

Despite numerous studies, the etiology of PCOS remains elusive. Insulin resistance is considered important to the pathophysiology of PCOS [3]. Some 50% to 70% of women with PCOS are insulin-resistant, suggesting that insulin resistance may even be an etiological factor for PCOS [4, 5]. Insulin resistance can be the result of lifestyle factors such as shiftwork, insufficient sleep or lack of exercise [6]. In particular, nurses working on night shifts are at risk of insulin resistance [7]. Lifestyle factors associated with insulin resistance can be interrelated, as shiftwork can lead to reduced time available for sleep and exercise [6]. The question arises as to whether these lifestyle factors, acting alone or in combination, can lead to changes in menstrual cycle length, clinical or biochemical hyperandrogenism and abnormal ovarian morphology, thereby resulting in the sub-phenotypes of PCOS.

Although shift work has been reported to be associated with menstrual cycle abnormalities, its relevance to the pathophysiology of PCOS is difficult to interpret, because both long and short menstrual cycle lengths can be induced by shiftwork. Studies involving USA nurses [8], Canadian poultry workers [9], and Taiwanese factory workers [10] suggest that women subjected to rotating shift work are more likely to have shorter or longer menstrual cycle lengths than those without shift work. Nurses working only fixed night shifts have been reported to have shorter menstrual cycle lengths (<25 days) [11]. Several studies have examined shift work in relation to reproductive hormone levels [12, 13], but little attention has been given to the associations of shiftwork, insufficient sleep or exercise (alone or in combination) with hyperandrogenism and ovarian morphology characteristic of PCOS. In particular, it is unknown whether these lifestyle factors can result in the pathophysiological changes of PCOS in healthy women. To fill these gaps, we examined associations of sleep length, lack of regular exercise, and shiftwork with clinical and the biochemical abnormalities typical of PCOS in a cohort of healthy women.

## Materials and Methods

### Study design, setting, and population

**Fig 1:**
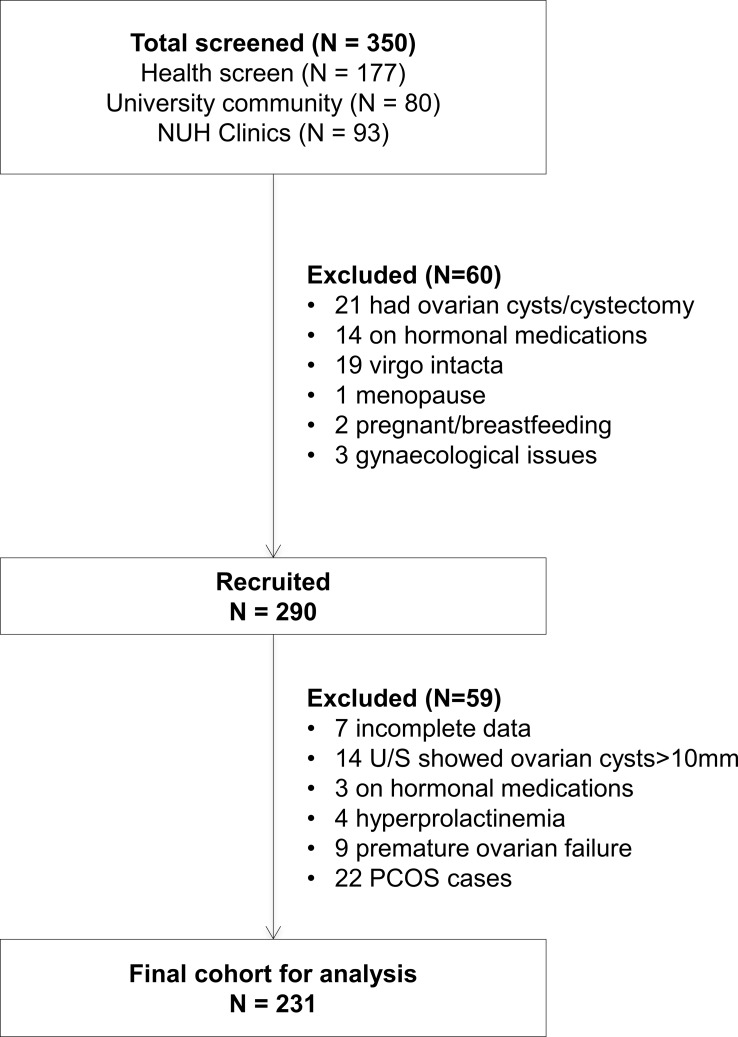
Flow chart showing study cohort selection.

We carried out a prospective cross-sectional study at the National University Hospital (NUH), Singapore, from 2011 to 2015. Our study recruited healthy women 21–45 years of age who participated in an annual health screen offered to all employees of the hospital (53%), plus healthy volunteers from the university community (28%) and healthy referral cases from the NUH Women’s Clinic (19%). Exclusion criteria were virgo intacta, breastfeeding, pregnancy, menopause or use of medications known to influence reproductive or metabolic function (such as lipid lowering and diabetic drugs) within 60 days of study entry. Women who self-reported having congenital adrenal hyperplasia, adrenal tumors, androgen-secreting tumors, thyroid disease, established diabetes, severe cardiovascular disease, hysterectomy and/or oophorectomy or established diagnosis of PCOS were excluded. In addition, women with hyperprolactinemia (>1000 mIU/L), ovarian cysts larger than 10mm, premature ovarian failure (anti-mullerian hormone (AMH) below 0.6 pmol/L, estradiol (E2) below 70 pmol/L or follicle stimulating hormone above 25.8 IU/L) and incomplete data were excluded from the final cohort for analysis (Fig 1). Biochemical assays on E2, FSH and prolactin to determine inclusion/exclusion was performed using assays and instruments from Beckman Coulter Inc. (Brea, CA, USA). Between menstrual cycle days 2 to 5 at 7:30–8:30 AM after an overnight fast, participants completed a health and lifestyle questionnaire, underwent physical examination (including assessment of hirsutism and measurements of height, weight, waist and hip circumferences), transvaginal ultrasound scan of the ovaries, and plasma sampling for androgenic and metabolic biomarkers. The study and consent procedure was approved by the National Health Group Domain Specific Review Board. Informed written consent was obtained from all participants.

### Lifestyle factors

Participants were asked to complete a questionnaire on lifestyle patterns that may affect reproductive function. Shift work, exercise and sleep duration were selected as the primary lifestyle factors in this study. We hypothesized that these factors would be associated with one another and would affect reproductive and metabolic outcomes. The frequency of shift work was recorded as no shift work, <3 times /month, between 4–10 times /month and >10 times /month. Sleep duration was recorded as <4 hours, between 4–6 hours, between 6–8 hours and >8 hours. Exercise was recorded as never, rarely, sometimes, regularly or competitively. Other lifestyle factors recorded were smoking (yes or no), alcohol consumption (never, occasionally, once a week, 2 to 3 times a week or every day) and stress levels reported on a scale of 1 (low stress) to 10 (extreme stress). In order to have sufficient numbers in each group for meaningful analysis, lifestyle factors were subsequently regrouped for analysis as shift work (yes/no), sleep length (<6 hours/ ≥ 6 hours), and alcohol consumption (never or occasionally/once a week or more). Short sleep is generally defined as < 6 hours [14]. For analysis of exercise, study women were regrouped into those who reported regular exercise (regularly or competitively) and those who did not (i.e., reported exercising never, rarely or sometimes). Dietary intake, current depression status, and sleep comorbidities (such as sleep apnea, restless legs syndrome, and difficulty falling or staying asleep) was not assessed.

### Menstrual cycle length

Study women were grouped into three categories of menstrual cycle lengths: <25 days, 25–34 days and ≥35 days. 25–34 day cycles were considered normal, <25 day cycles were considered short and ≥35 day cycles were considered long [15].

### Hyperandrogenism

#### Clinical

Modified Ferriman–Gallwey scoring (mFG) for hirsutism was performed by two clinicians, using printed standard photographs as reference [16]. To standardize results, initial assessments were made independently, and the scores were then compared for inter-reliability. This process was repeated for 15 study women until a consensus in scoring was reached between the two clinicians.

#### Biochemical

Testosterone was detected by competitive binding immunoassays and sex hormone binding globulin (SHBG) was measured by a sandwich immunoassay (Beckman Coulter Inc). Free Androgen Index (FAI) was calculated as the percentage ratio of total testosterone to SHBG values.

### Ovarian morphology and function

Transvaginal ultrasound measurements were performed in real time with a Voluson E8 machine and a 6–12 MHz transducer as described previously [15]. Measurements were performed in real time. Subjects with ovarian follicles or cysts >10 mm were asked to return in the next cycle for repeat evaluation. Subjects with at least one follicle with a mean diameter >10 mm were excluded to avoid spuriously high ovarian volume and hormonal effects from the emergent dominant follicle. The AFC was the number of antral follicles visible on ultrasound in both ovaries.

#### Anthral follicle counts (AFC)

All visible follicles in both ovaries were counted, and diameters of follicles were determined as the mean of two diameters (longitudinal and antero-posterior). Women with ovarian follicles or cysts >10 mm were asked to return in the next cycle for repeat evaluation. Women with at least one follicle with a mean diameter >10mm were excluded to avoid spuriously high ovarian volume and hormonal effects from the emergent dominant follicle.

#### Ovarian volume

After determining the longest medial axis of the ovary, the length and height of the ovaries were measured, and the probe was then turned to measure the width. Ovarian volume was calculated using the formula for the volume of a prolate ellipsoid: V = 0.523 x length x height x width. The measure used for analysis was the mean of the left and right ovarian volumes.

#### Anti-mullerian hormone

*Anti-mullerian hormone (AMH)*, a surrogate measure of ovarian function, was measured with the Gen II ELISA assay, following the recommended premix procedure, and using the semi-automated programmed Evolis immunoassay system (Bio-Rad, Hercules, CA, USA)

### Metabolic outcomes

Body mass index (BMI), defined as the ratio of body weight to height squared, and waist to hip ratio (WHR) were determined. Levels of glucose, insulin and lipids were measured using fasted serum. Glucose, insulin and lipids (triglycerides, total cholesterol, HDL, and LDL) were measured using assays and instruments from Beckman Coulter Inc. The homeostasis model assessment-estimated insulin resistance (HOMA-IR), an indicator of insulin resistance, was calculated as (glucose x insulin)/22.5 [17]. We defined the women in the highest quartile of the HOMA-IR distribution as insulin resistant. This method has been shown to be a reliable alternative to the euglycemic hyperinsulinemic clamp in defining insulin resistance [18]. The upper quartile cut-off value for HOMA-IR was 1.96 in our study sample. Metabolic syndrome was defined using the International Diabetes Federation criteria for South Asian women.

All assay performance characteristics were determined contemporaneously and shown in S1 Table.

### Statistical analysis

Associations between shift work, exercise or sleep duration with participants’ reproductive and metabolic outcomes were examined using Student’s t-test for continuous variables with a normal distribution (average ovarian volume, AMH, testosterone, SHBG, BMI, WHR, average diastolic blood pressure, cholesterol, LDL and HDL), the Mann-Whitney U test for continuous variables with a non-normal distribution (anthral follicular count, mFG score, FAI, average systolic blood pressure, fasting triglycerides, fasting glucose, fasting insulin and HOMA-IR) and the chi-square test for the categorical variable of menstrual cycle length. Multivariable linear regression and logistic regression were used, where appropriate, to adjust for confounding factors (age, race (Chinese or non-Chinese), education (university or below university), stress levels (scale of 1 to 10), having given birth in the last 5 years and BMI) that could affect the associations of sleep length or exercise with the reproductive and metabolic outcomes. Only 12 women were smokers, 2 women consumed alcohol once a week or more, and 10 women had a previous medical history of depression and/or anxiety, so these variables were not considered further. Coffee consumption was not included as a potential confounder, as the temporal sequence between coffee consumption and sleep is unclear. All statistical analyses were performed using SPSS software 20.0 (SPSS, Chicago, Illinois). The area-proportional Venn diagram was generated using EulerAPE v3.0 [19].

## Results

Characteristics of the study women (n = 231) are shown in Table 1. Average age was 31.6 years, 73% were of Chinese ethnic origin, 64% had at least a university education, and 62% had executive or professional occupations and 21% gave birth in the last 5 years (i.e., had a young child to care for). All study women had menstrual cycle lengths of <60 days and there were no cases of amenorrhea or grossly irregular menses.

**Table 1:** Characteristics of study women (N = 231).

Characteristic	Number (%)
Age*	31.6 (5.6)
Race	
Chinese	170 (73.6)
Non-Chinese	61 (26.4)
Education	
Below university	81 (35.1)
University	150 (64.9)
Occupation	
Executive	35 (15.2)
Professional	110 (47.6)
Technical support	11 (4.8)
Sales	6 (2.6)
Clerical/admin support	18 (7.8)
Service	17 (7.4)
Self-Employed	7 (3.0)
Unemployed	16 (6.9)
Others	11 (4.8)
Medical history of depression and/or anxiety	
Yes	10 (4)
No	221 (96)
No of hours of sleep	
<6 hours	55 (23.8)
> = 6 hours	176 (76.2)
Shift work	
Yes	57 (24.7)
No	174 (75.3)
Coffee consumption	
> = 1 cup/day	69 (29.9)
<1 cup/day	162 (70.1)
Stress levels*	5.3 (1.8)
Gave birth in last 5 years*	
Yes	48 (20.8)
No	183 (79.2)
Regular Exercise	
Yes	65 (24.2)
No	175 (75.8)
Smoking	
Yes	12 (5.2)
No	219 (94.8)
Alcohol consumption	
Never/Occasionally	229 (99.1)
Once a week or more	2 (0.9)

* Mean (SD)

**Fig 2:**
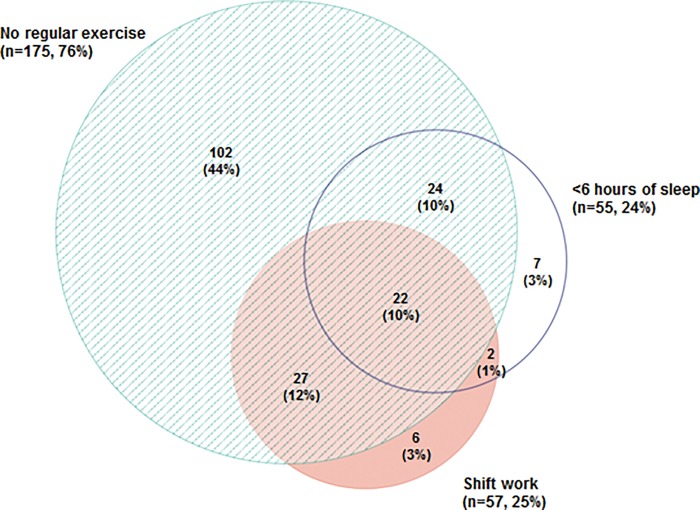
Venn diagram showing the prevalence and overlap of lifestyle risk factors. Numbers (and percentages of total study sample) refer to study women with one or more of the three lifestyle risk factors alone and in combination.

Fig 2 shows the prevalence and overlap of these risk factors in the study women. The majority (n = 190, 82%) of study women reported at least one of the three lifestyle risk factors under study: shift work, no regular exercise or short sleep duration.

Twenty-four percent of the study women reported doing shift work. Shift work was not associated with any of the reproductive outcomes. Shift work was associated with increased WHR, but not with any of the other metabolic outcomes analyzed (Table 2).

**Table 2:** Lifestyle factors and reproductive and metabolic outcomes.

	Shiftwork			No Regular Exercise			Sleep hours		
	No	Yes	Significance	No	Yes	Significance	> = 6 hours	<6 hours	Significance
***Reproductive***									
	*Frequency (%) *								
Menstrual cycle length (days)									
<25	9 (5.2)	7 (12.3)		1 (1.8)	15 (8.6)		8 (4.5)	8 (14.5)	**
25–34	119 (68.4)	36 (63.2)		40 (71.4)	115 (65.7)		127 (72.2)	28 (50.9)	
> = 35 days	46 (26.4)	14 (24.6)		15 (26.8)	45 (25.7)		41 (23.3)	19 (34.5)	
	*Mean (SD)*								
Avg ovarian volume	5.09 (2.28)	5.48 (2.50)		5.10 (2.16)	5.22 (2.40)		5.19 (2.30)	5.19 (2.49)	
Anthral follicle count	15.8 (11.7)	16.9 (11.1)		15.1 (10.1)	16.4 (11.9)		15.7 (10.4)	17.2 (14.7)	
AMH (pmol/L)	41.9 (34.1)	47.6 (41.8)		40.1 (30.5)	44.3 (37.8)		43.0 (33.8)	44.3 (43.2)	
mFG score	1.09 (1.39)	1.09 (1.78)		1.21 (1.39)	1.05 (1.52)		1.09 (1.48)	1.07 (1.52)	
SHBG (nmol/L)	60.5 (31.2)	54.8 (23.8)		62.5 (32.6)	57.9 (28.6)		61.1 (31.2)	52.6 (22.8)	*
Testosterone (nmol/L)	1.32 (0.71)	1.25 (0.65)		1.45 (0.74)	1.25 (0.67)		1.31 (0.70)	1.28 (0.67)	
FAI	3.19 (3.49)	3.06 (2.58)		3.24 (3.71)	3.13 (3.15)		3.14 (3.46)	3.22 (2.69)	
	*Mean (SD)*								
***Metabolic***									
BMI	22.5 (4.0)	23.1 (4.2)		22.1 (3.3)	22.8 (4.3)		22.1 (3.6)	24.2 (5.1)	**
WHR	0.77 (0.06)	0.80 (0.06)	**	0.76 (0.06)	0.78 (0.06)	*	0.78 (0.06)	0.79 (0.06)	
Average systolic blood pressure (mm Hg)	111 (14)	112 (12)		113 (15)	112 (13)		111 (12)	115 (17)	
Average diastolic blood pressure (mm Hg)	67.5 (9.8)	68.0 (9.3)		66.8 (8.4)	67.9 (10.0)		67.0 (9.4)	69.5 (10.3)	
Cholesterol (mmol/L)	4.65 (0.76)	4.72 (0.84)		4.71 (0.80)	4.65 (0.77)		4.62 (0.76)	4.79 (0.82)	
Triglycerides (mmol/L)	0.91 (0.46)	0.96 (0.73)		0.93 (0.54)	0.92 (0.54)		0.89 (0.46)	1.01 (0.72)	
HDL (mmol/L)	1.50 (0.34)	1.50 (0.44)		1.49 (0.30)	1.50 (0.39)		1.51 (0.36)	1.48 (0.37)	
LDL (mmol/L)	2.72 (0.66)	2.76 (0.74)		2.75 (0.71)	2.72 (0.67)		2.70 (0.66)	2.82 (0.74)	
Glucose (mmol/L)	4.65 (0.45)	4.57 (0.40)		4.65 (0.43)	4.62 (0.45)		4.62 (0.44)	4.65 (0.44)	
Insulin (mU/L)	7.96 (6.74)	7.90 (4.37)		6.14 (3.85)	8.52 (0.63)	**	7.19 (5.23)	10.4 (8.3)	***
HOMA-IR	1.68 (1.53)	1.62 (0.97)		1.29 (0.88)	1.78 (1.52)	**	1.51 (1.30)	2.13 (1.63)	***
	*Frequency (%) *								
Insulin resistance									
No	132 (75.9)	42 (73.7)		47 (83.9)	127 (72.6)		143 (81.2)	31 (56.4)	***
Yes	42 (24.1)	15 (26.3)		9 (16.1)	48 (27.4)		33 (18.8)	24 (43.6)	
Metabolic Syndrome									
No	167 (96.0)	54 (94.7)		51 (91.1)	170 (97.1)		171 (97.2)	50 (90.9)	
Yes	7 (4.0)	3 (5.3)		5 (8.9)	5 (2.9)		5 (2.8)	5 (9.1)	

* P<0.05

** P<0.01

*** P<0.001

Seventy-six percent of study women did not exercise regularly. No differences were observed in menstrual cycle length or in ovarian or androgen outcomes in women who exercised regularly compared to those who did not (Table 2). Women who did not exercise regularly had significantly higher mean WHR (0.78 ± 0.06 vs 0.76 ± 0.06, p = 0.02), but did not have a significantly higher mean BMI. Lack of regular exercise was associated with significantly higher mean fasting insulin (difference in means = 2.3 mU/L; 95% CI, 0.5 to 4.1) and HOMA-IR (difference in means = 0.49; 95% CI, 0.09 to 0.90) levels but not with the risk of insulin resistance, after adjusting for potential confounders and the other lifestyle factors (shift work and sleep length).

Twenty-three percent of the study women reported fewer than 6 hours of sleep. Menstrual cycle length was significantly associated with sleep length (χ^2^ = 10.9, p = 0.004, see Table 2). After adjustment for confounders and the other lifestyle factors (exercise and shift work) women who reported less than 6 hours of sleep had significantly higher odds of an abnormal cycle length (short or long) than women with 6 or more hours of sleep (OR = 2.1; 95% CI, 1.1 to 4.2). In multinomial logistic regression analyses adjusting for potential confounders and exercise other lifestyle factors (exercise and shift work), short sleep duration was significantly associated with having short cycles (vs normal cycles) (OR = 3.7; 95% CI,1.1 to 12.7) and non-significantly associated with having longer cycles (vs normal cycles) (OR = 1.7, 95% CI, 0.8 to 3.7) compared to those who had 6 or more hours of sleep. No significant interactions (synergistic effects) were observed between sleep and shift work or exercise and menstrual cycle length. SHBG was significantly lower in women who reported less than 6 hours of sleep (52.6 ± 22.8 vs 61.1 ± 31.2 nmol/L, p = 0.03), but no changes were observed in the other ovarian and androgenic measures.

With respect to metabolic outcomes, women who reported fewer than 6 hours of sleep had a significantly higher mean BMI (24.2 ± 5.1 vs 22.1 ± 3.6, p = 0.008), fasting insulin levels (10.4 ± 8.3 vs 7.2 ± 5.2) mU/L, P<0.001) and HOMA-IR (2.1 ± 1.6 vs 1.5 ± 1.3, p<0.001). After adjustment for confounders and other lifestyle factors, short sleep duration remained significantly associated with increased fasting insulin levels (difference in means = 2.1; 95% CI, 0.3 to 4.0 mU/L) and higher odds of insulin resistance (OR = 2.6; 95% CI, 1.2 to 5.8) and non-significantly associated with HOMA-IR (difference in means = 0.40; 95% CI, -0.02 to 0.82) compared to women who reported 6 or more hours of sleep. No significant interactions were observed with shift work or insufficient exercise for fasting insulin levels or HOMA-IR.

## Discussion

The lifestyle factors we examined (shift work, short sleep duration and regular exercise) were not significantly associated with any ovarian (ovarian volume, anthral follicle count, serum AMH) or androgenic measures (mFG score, serum testosterone) that define PCOS. Analyses of women who reported fewer than 6 hours of sleep, those who reported no regular exercise, and those who performed shift work revealed no significant associations with the ovarian and androgenic parameters studied. Nor did shift work or insufficient exercise have an impact on menstrual cycle length. Thus, our findings do not support the hypothesis that shift work or lack of exercise results in the clinical or biochemical characteristics typical of PCOS. These findings differ from those reported in previous studies reporting that night shift workers have lower mean serum testosterone levels than day shift workers [13], and that women with more than 20 months of rotating shift work were at risk of short (<21 days) or long (>40 days) menstrual cycles [8].

We observed that short sleep duration, rather than shiftwork, was significantly associated with changes in menstrual cycle length. In women who reported fewer than 6 hours of sleep, 15% had short cycle lengths and 35% had long cycle lengths. In contrast, among women who reported 6 or more hours of sleep, only 5% had short cycle lengths and 23% had long cycle lengths. After adjustment for confounders, women reporting fewer than 6 hours of sleep had a 3.9-fold increased risk of having short menstrual cycles (less than 25 days).

Recent findings suggest that there are circadian rhythm and sleep disturbances in women with diagnosed PCOS [20, 21]. We also observed that women with less than 6 hours of sleep had a 1.7-fold increased risk of having long menstrual cycles (more than 35 days) typical of PCOS, although this association did not reach statistical significance. The small number of women with long cycles in our cohort limited our statistical power to detect such an association.

Interestingly, we found that a lack of sleep is significantly associated with shorter menstrual cycles. It is tempting to speculate that disruption of pulsatile FSH and LH secretion associated with disordered sleep [12, 21, 22] may adversely affect luteinization of granulosa cells, resulting in luteal phase defect [23], which can lead to short menstrual cycles. However, short sleep duration was not significantly associated with any changes in reproductive hormone levels (FSH, LSH, estrogens, androgens) or ovarian variables (volume, anthral follicle counts or AMH).

Strong experimental evidence [24] indicates that recurrent nights of insufficient sleep can cause insulin resistance and diabetes in healthy adults. This has also been confirmed in epidemiological studies [25]; nurses working night shifts have been noted to have a five-fold increased risk of metabolic syndrome [7]. Short sleep duration had also been associated with a higher risk of the pre-diabetic state when compared with individuals with 7 or more hours of sleep per night [26]. Such an association may be attributable to increased concentrations of insulin and HbA1c in individuals with short sleep duration, which may be a consequence of their higher BMI [27]. Insufficient or disordered sleep has been associated with an increased risk of type 2 diabetes [26, 28].

Consistent with the findings of previous studies, we observed that short sleep duration is significantly associated with metabolic abnormalities, including higher BMI, raised fasting insulin levels, and increased risk of insulin resistance. The associations between sleep and fasting insulin levels and insulin resistance remained significant even after adjustment for BMI and other confounders. In particular, women who reported fewer than 6 hours of sleep had a 2.6 fold higher adjusted risk of insulin resistance compared to women who reported 6 or more hours of sleep. Women with short sleep duration were also at increased risk of short menstrual cycles. Given our findings, the link between menstrual cycle length, insulin metabolism and ovarian function should be pursued in further studies.

An important strength of our study is that it is not based on a referred sample of symptomatic women, but on healthy hospital workers and volunteers from a university community who agreed to undergo detailed clinical assessment for hyperandrogenism, ultrasound examination of ovarian morphology and measurement of reproductive and metabolic hormone levels. A limitation of our study includes its recruitment from a single center, which might compromise the generalizability of our results. In addition, information on sleep duration was based on recall, and we were unable to perform polysomnography to determine other aspects of sleep, such as the quality of sleep and duration of rapid eye movement sleep. Although we have adjusted for as many factors that could affect the associations of sleep length or exercise with the reproductive and metabolic outcomes, there is the possibility of residual confounding due to factors which we lack information on, such as diet and current depression status. Also, there may be a possible over-reporting of exercise due to a lack of accurate actigraphy data, but any such over-reporting should not differ according to sleep duration or quality and therefore would bias any observed association towards the null.

In conclusion, our results suggest that women with insufficient sleep are at increased risk of insulin resistance and menstrual disturbances. Sufficient sleep may be important to protect women against ovulatory dysfunction, possible fertility problems and insulin resistance. Larger epidemiologic studies are required to confirm our results, and mechanistic investigations should help delineate the pathways underlying the associations we observed.

## Supporting Information

S1 Table Assay performance characteristics. (DOCX)
