# Supplementary material for: Sleep Duration, Exercise, Shift Work and Polycystic Ovarian Syndrome-Related Outcomes in a Healthy Population: A Cross-Sectional Study
Source: PLoS One. 2016 Nov 21;11(11):e0167048. doi: 10.1371/journal.pone.0167048 (PMC5117764; doi:10.1371/journal.pone.0167048)
Supplement: S1 Table — (DOCX) [file pone.0167048.s001.docx]

| **Supplemental Table 1.** Assay performance characteristics | | | |
| --- | --- | --- | --- |
| Variable (units) | Concentration Range | Intra-assay CV (%) | Inter-assay CV (%) |
| Glucose (mmol/L) | 2.1 - 24.4 | 0.6 - 1.6 | 0.8 - 2.0 |
| Cholesterol (mmol/L) | 3.62 - 9.91 | 0.3 - 0.8 | 0.6 - 1.2 |
| Triglycerides (mmol/L) | 0.60 - 5.31 | 0.5 - 1.0 | 0.7 - 1.3 |
| HDL (mmol/L) | 0.62 - 2.16 | 0.6 - 0.8 | 1.2 - 1.6 |
| Insulin (mIU/L) | 1.8 - 246.4 | 0.0 - 5.1 | 3.4 - 6.4 |
| Estradiol (pmol/L) | 239 - 15059 | 2.1 - 9.3 | 5.6 - 9.8 |
| LH (IU/L) | 5.8 - 93.6 | 0.9 - 3.9 | 3.7 - 6.7 |
| FSH (IU/L) | 2.5 - 119.5 | 2.1 - 4.0 | 3.6 - 7.1 |
| Prolactin (mIU/L) | 66 - 3726 | 2.5 - 5.1 | 3.3 - 7.0 |
| Testosterone (nmol/L) | 1.06 - 29.35 | 2.3 - 5.7 | 2.6 - 11.0 |
| SHBG (nmol/L) | 16 - 164 | 1.8 - 3.7 | 3.1 - 5.9 |
| Anti-Mullerian Hormone (pmol/L) | 11.95 - 56.53 | 3.5 - 4.8 | 5.5 - 7.2 |
